# Editorial: Linking knowledge across borders: science diasporas and global south development

**DOI:** 10.3389/frma.2026.1979687

**Published:** 2026-09-15

**Authors:** Luisa F. Echeverría-King, Kleinsy Bonilla, Branislav Pantović, Efraín Bámaca-López, Ana Maria Carneiro, Derya Buyuktanir Karacan

**Affiliations:** 1 Universidad Simon Bolivar, Barranquilla, Colombia; 2 Observatorio Económico Sostenible, Universidad del Valle de Guatemala, Guatemala City, Guatemala; 3 DiploCientifica, Viedma, Argentina; 4 School of Journalism, Faculty of Humanities, University of Santiago de Chile - USACH, Santiago, Chile; 5 Núcleo de Estudos de Políticas Públicas, Universidade Estadual de Campinas, Campinas, Brazil; 6 The Institute for International Science and Technology (IISPT), Elliott School of International Affairs, George Washington University, Washington, DC, United States

**Keywords:** brain circulation, diaspora engagement, Global South, migration, science diplomacy, scientific collaboration, scientific diaspora, Technology and Innovation (STI)

This Research Topic, *Linking Knowledge Across Borders: Science Diasporas and Global South Development*, originates from an issue that is particular to the Global South: countries already constrained in their efforts to build scientific and technological capabilities are often among the most affected by the international outflow of highly skilled talent (Docquier et al., 2007). Namely, many scientific systems in the Global South operate with inadequate or unstable funding, uneven access to research infrastructure, limited career opportunities, and restricted influence over the networks and funding structures that shape international research agendas and opportunities (OECD, 2023, p. 193; Schneider et al., 2023). In these circumstances, the loss of highly qualified professionals could be logical and can have disproportionately severe consequences due to less resilient scientific and technological systems (Di Maria and Lazarova, 2012).

However, the migrational process does not conclude when scientists relocate. Scientific capabilities dispersed across different geographical areas may remain interconnected or could return. In both cases, knowledge, expertise, access to resources, and professional relationships can circulate across countries' borders (Meyer and Wattiaux, 2006). The developmental value of scientific diasporas therefore lies not only in the expertise they embody, but also in the institutional capacity to identify, connect, and sustain engagement with these transnational communities, including through diplomatic missions and foreign-affairs institutions (Bonilla, 2021; Bonilla and Serafim, 2021; Ezekiel and Mauduit, 2023). These possibilities raise a complex question that ultimately motivated the development of this Research Topic: under what conditions does the scientific diaspora serve as a resource for countries of origin and their institutions, and in which situations does it merely result in a geographical redistribution of already limited scientific capacity? The answer is not straightforward. A diaspora does not automatically become an asset, and successful policy models cannot be universally applied (Echeverría-King et al., 2025).

This combination of urgency, potential, and unresolved questions explains the significant interest generated by the call for contributions. Scholars and practitioners from diverse regions, disciplines, policy environments, and backgrounds addressed the subject, indicating that scientific diaspora engagement is an increasingly widespread concern throughout the Global South, rather than an issue confined to a few migrant-sending countries.

This Research Topic brings together 18 contributions encompassing Original Research articles, Perspectives, Brief Research Reports, Policy and Practice Reviews, Community Case Studies, and Conceptual Analyses. Together, these manuscripts propose viewing scientific diasporas not simply as communities of expatriate researchers, under different institutional conditions and contexts, but instead portray them as *transnational knowledge infrastructures* that connect institutions, disciplines, countries, and policy arenas. Likewise, some study migration preferences, repatriation, identity, institutional affiliation, or potential engagement. Drawing on evidence from Latin America—including Venezuela, Guatemala, Argentina, Honduras, Colombia, Brazil, Costa Rica, and regional studies—alongside contributions from Türkiye, Mexico, and the African scientific diaspora in the Nordic region, this collection illustrates both the diversity of diaspora experiences and the common structural challenges shaping scientific mobility and international collaboration across the Global South.

## Scientific diasporas as transnational actors for development and governance

The Venezuelan case illustrates this transformation from multiple complementary perspectives. In *The Venezuelan Scientific Diaspora as a Transnational Actor: Knowledge Circulation, STI Cooperation, and Development*, Echeverría-King et al. examine how Venezuelan scientists dispersed across different world regions continue to contribute to science, technology, and innovation despite prolonged national institutional fragility. Drawing on survey data and participatory focus groups, the study highlights the diaspora's willingness and capacity to engage in scientific cooperation, identifies barriers to participation, and proposes actionable strategies for strengthening regional Science, Technology and Innovation (STI) collaboration. Rather than depicting the diaspora as a consequence of crisis alone, the article positions it as a potential strategic actor capable of sustaining scientific collaboration and fostering resilience across national and regional innovation ecosystems.

This perspective is further expanded by *Rethinking Scientific Diaspora Engagement Models In Fragile Contexts: The Venezuelan Case*, where Pantović et al. argue that prevailing engagement frameworks are largely designed for countries with relatively stable STI systems and therefore inadequately address contexts characterized by institutional fragility. Their analysis proposes more flexible and context-sensitive engagement models that recognize scientific diasporas as autonomous non-state actors capable of maintaining international scientific linkages even when domestic institutions experience prolonged disruption. Together, both Venezuelan studies contribute to a broader rethinking of diaspora engagement by demonstrating that fragility should not be interpreted solely as a barrier to engagement; rather, it changes the conditions under which engagement must be designed, calling for more flexible, distributed, and context-sensitive institutional arrangements.

*Brain Linkage for good governance: the need for guatemalan diaspora expertise* (Bonila, Reyes et al.) broadens the discussion by positioning Guatemala's scientific and professional diaspora as a strategic resource for strengthening governance and evidence-informed policymaking. Bonila, Reyes et al. argue that the potential contribution of diaspora expertise extends beyond academic collaboration, emphasizing its role in institutional development, evidence-informed policymaking, and governance.

Likewise, *Integration between the Knowledge diaspora and their country of origin through institutional affiliations: a case study for policy development* proposes a framework based on long-term institutional affiliation. Using Argentina as a case study, Rotstein argues that sustained institutional integration offers an alternative to conventional repatriation and linkage models.

## From brain drain to brain circulation: mobility, identity, and structural inequalities

This conceptual shift is explicitly examined in *Brain drain or brain circulation? A perspective from Latin American scientists*, where Freire-Fierro et al. combine conceptual analysis with survey evidence from Latin American researchers to investigate how structural asymmetries continue to shape international scientific collaboration. The study identifies persistent inequalities in authorship, funding, language, and agenda-setting that favor Global North institutions, while suggesting that diaspora researchers may be particularly well-positioned to support more equitable international partnerships through knowledge brokerage and collaborative networks. Importantly, the authors argue that the transformative potential of brain circulation remains constrained unless broader structural inequities within the global research system are addressed.

*From brain drain to brain circulation: evidence from women scientists in OWSD honduras national chapter* (Cabrera-Medina et al.) examines the gendered dimensions of scientific mobility. Cabrera-Medina et al. show how the OWSD Honduras National Chapter fosters brain circulation by connecting women scientists through collaboration, mentoring, and knowledge exchange, highlighting the value of gender-responsive scientific networks in resource-constrained contexts.

While institutional factors receive considerable attention throughout the collection, *Post-material values and brain drain: how value transformation shapes the migration mindset among highly educated South Asians?* (Udayanga) introduces a complementary sociological perspective. Udayanga argues that migration decisions cannot be explained exclusively through economic incentives or labor market opportunities. Instead, highly educated professionals often migrate because of deeper value transformations associated with autonomy, professional recognition, social justice, and quality of life. This perspective challenges conventional return-oriented policies by suggesting that effective diaspora engagement must also acknowledge the cultural, institutional, and normative conditions that shape long-term relationships between highly skilled migrants and their countries of origin. Importantly, where migration reflects a deeper mismatch between individual aspirations and prevailing institutional or social environments, incentives for return, and potentially for sustained engagement, may themselves be weakened.

*Repatriation, identity, and organisational adaptation: a case study on reverse brain drain and socio-psychological drivers in Turkey's energy sector* (Durmuşkaya et al.) shifts the focus from migration decisions to the post-return experience. Durmuşkaya et al. show that successful repatriation depends not only on attracting talent back but also on organizational environments that facilitate integration, identity, and long-term retention. Their case study highlights how conditions and mismatches between expertise and professional roles can shape returnees' experiences. The study expands discussions of reverse brain drain by emphasizing the institutional and psychological dimensions of knowledge reintegration.

*Between Linkage and Return: The Strategies of the Knowledge Brazil Program for the Scientific Diaspora* (Cavasin and Carneiro) examines how Brazil combines repatriation, linkage, and knowledge circulation within a national diaspora policy. Cavasin and Carneiro highlight both the opportunities and the challenges of sustaining long-term institutional engagement, since the program remains strongly oriented toward return and retention than toward sustained linkage with researchers abroad. The Brazilian case therefore illustrates the expansion of diaspora engagement mechanisms and the continuing institutional challenge of moving beyond predominantly repatriation-centered approaches, despite the growing emphasis on knowledge circulation.

In their contribution, *From Brain Drain to Geopolitical Talent Competition: Scientific Diasporas and the Future of Global South Development*, Karacan and Takaki argue that the traditional brain-drain framework no longer captures the multidirectional, networked character of contemporary scientific mobility. Drawing on scholarship on scientific diasporas, science diplomacy, and global talent competition, the authors position highly skilled migrants as transnational development partners embedded in a broader “network of networks” linking diaspora scientists, research institutions, and policy actors. The paper traces how intensifying geopolitical competition for talent, shifting U.S. and European science and immigration policies, and the rise of scientific hubs beyond the West are reshaping this landscape, with particular attention to the Global South, South–South cooperation, and reverse brain drain. It closes with tiered policy recommendations for Global South governments, developed countries, and international organizations, and introduces “geotalent”—a term the authors propose to capture how geography, geopolitics, geo-economics, and environmental change jointly shape today's flows of scientific talent.

## Building knowledge infrastructures: mapping, networks, and policy innovation

*Mapping and engaging the Costa Rican scientific diaspora: experiences from over a decade implementing the hipatia.cr portal* (Duran-Monge and Santos) presents long-running scientific diaspora initiatives in Latin America. Drawing on more than a decade of implementation, Duran-Monge and Santos demonstrate how systematic mapping, digital platforms, and continuous data collection can support evidence-informed policymaking while creating sustainable institutional mechanisms for diaspora engagement.

Complementing the Costa Rican experience, *Diaspora contributions: evidence from the outliers network building in Guatemala* (Sáenz et al.) examines how the Outliers initiative connects members of Guatemala's scientific and professional diaspora with local innovation actors. Sáenz et al. show that community-based model networks can foster trust, cross-sector collaboration, and knowledge exchange, highlighting the value of social infrastructures in complementing formal institutional engagement.

The role of systematic characterization is further examined in *Guatemalan scientific and professional diaspora in spain: an initial characterization* (Bonilla, Romero-Oliva et al.). Bonilla, Romero-Oliva et al. provide an initial empirical characterization of this diaspora, documenting its demographic characteristics, migration trajectories, collaborative interests, and institutional challenges. Beyond generating descriptive evidence, the study identifies structural barriers—including limited visibility, fragmented institutional coordination, and insufficient engagement mechanisms—that continue to constrain the contribution of highly skilled Guatemalans abroad. Importantly, the article also draws attention to inequalities within scientific mobility itself, noting the underrepresentation of Indigenous populations among highly skilled migrants and calling for more inclusive approaches to diaspora engagement.

## Science diplomacy, knowledge diplomacy, and addressing global challenges

Environmental governance provides one of the clearest examples of this expanding role. In *Scientific diasporas in global biodiversity governance: brain circulation, linkage, and knowledge brokerage in Colombia*, Prieto et al. examine how members of the Colombian scientific diaspora contribute to biodiversity governance through international collaboration, technology transfer, capacity building, and policy advice. Drawing on evidence generated around COP16, the study develops an exploratory typology of diaspora engagement mechanisms, demonstrating that diaspora scientists could contribute not only through academic research but also through co-production with societal actors, institutional partnerships, and science-policy interfaces. The findings reinforce the idea that scientific diasporas serve as valuable intermediaries linking global environmental commitments to national implementation efforts, while also pointing to the institutional conditions required for this brokerage role to translate into sustained policy engagement.

A complementary perspective is offered in *Connecting knowledge and action: recommendations to leverage Guatemala's scientific diaspora to address water management challenges* (Franco et al.). Franco et al. argue that diaspora expertise could strengthen science-policy interfaces by connecting researchers, policymakers, and other stakeholders to address Guatemala's water governance challenges.

The international dimensions of scientific cooperation are explored from a different angle in *Science diplomacy in the Global South: a case study of Brazilian participation in the PLATO mission* (Gueter et al.). Gueter et al. analyze Brazil's participation in the European Space Agency's PLATO (PLAnetary Transits and Oscillations of stars) mission to illustrate how large-scale international scientific collaborations can simultaneously advance scientific objectives and wider diplomatic engagement. Their findings demonstrate that scientific cooperation can evolve into broader processes of institutional relationship-building, international visibility, and science diplomacy while also revealing the structural asymmetries and governance constraints that continue to shape Global South participation in major international research initiatives.

The collection further expands the conceptual boundaries of the field through *Diaspora-driven knowledge diplomacy: rethinking transnational education through the CIESDEMEX model in the Global South* (Romero et al.). Romero et al. argue that diaspora engagement, transnational education, and knowledge diplomacy should not be understood as separate policy domains but as interconnected dimensions of internationalization. Building upon the CIESDEMEX (Consorcio de Instituciones de Educación Superior para el Desarrollo Educativo de las Personas Mexicanas en el Exterior) model, the authors propose an integrated conceptual framework in which higher education institutions, diaspora networks, and international cooperation mechanisms jointly facilitate sustained knowledge circulation. In doing so, the article operationalizes knowledge diplomacy as a governance framework capable of aligning education, scientific collaboration, and development objectives across multiple institutional levels.

Finally, *The scientific African diaspora in the Nordic region: policy perspectives on strengthening Africa–Nordic technology collaboration* (Afrifa and Del Valle) is another article that expands the collection beyond Latin America. Afrifa and Del Valle highlight how the African scientific diaspora can foster reciprocal innovation, institutional partnerships, and capacity building, reinforcing the role of scientific diasporas as bridge-builders in North–South cooperation. Their analysis also emphasizes that realizing this role requires sustained institutional support and appropriate engagement mechanisms.

As Guest Editors, we hope this Research Topic serves not only as a collection of studies but also as an invitation to advance the global conversation on the role of scientific diasporas in science, technology, innovation, and sustainable development. Taken together, these contributions suggest that scientific diaspora engagement should neither be reduced to physical return nor assumed to generate developmental benefits automatically. The contributions presented here demonstrate the diversity of contexts, approaches, and policy experiences that are shaping this rapidly evolving field while highlighting the need for more comparative, interdisciplinary, and practice-oriented research. At the same time, the collection shows that scientific mobility is itself shaped by structural inequalities in access to funding, infrastructure, recognition, language, and opportunities for participation and agenda-setting.

Scientific diasporas can therefore act as brokers, connectors, and institutional intermediaries, but they cannot substitute for strong and inclusive scientific systems. Additionally, the diversity of articles shows that scientific diasporas comprise heterogeneous communities whose relationships with scientific systems and societies are influenced by distinct trajectories, policies, organizational forms, and levels of connection. Maintaining these relationships requires both continuity and adaptability across various forms of participation (Echeverría-King et al., 2025; Pombo, 2025), while also recognizing scientific diasporas as actors in their own right rather than solely as resources to be mobilized (Hernández Mondragón and Cuapio, 2025).

We encourage researchers, policymakers, higher education institutions, funding agencies, and diaspora organizations to engage with these findings, expand the evidence base, and translate knowledge into innovative policies and collaborative initiatives. As global challenges increasingly require cross-border cooperation, scientific diasporas can play a distinctive role in connecting knowledge, institutions, and people across regions. We hope this collection stimulates new research, partnerships, and policy dialogue that continue to strengthen scientific collaboration and knowledge circulation across the Global South and beyond.
